# Seasonal fluctuation and alternative host plants of vegetable crop-infesting tephritids in non-vegetable growing areas in South Sudanese zone of Burkina Faso

**DOI:** 10.1093/jisesa/ieae047

**Published:** 2024-05-04

**Authors:** Issaka Zida, Alizèta Sawadogo, Souleymane Nacro

**Affiliations:** Centre National de la Recherche Scientifique et Technologique, Institut de l’Environnement et de Recherches Agricoles, Laboratoire d’Entomologie Agricole, Station de Farako-Bâ, BP 910 Bobo-Dioulasso, Burkina Faso; Centre National de la Recherche Scientifique et Technologique, Institut de l’Environnement et de Recherches Agricoles, Laboratoire d’Entomologie Agricole, Station de Farako-Bâ, BP 910 Bobo-Dioulasso, Burkina Faso; Centre National de la Recherche Scientifique et Technologique, Institut de l’Environnement et de Recherches Agricoles, Laboratoire d’Entomologie Agricole, Station de Kamboinsé, BP 476 Ouagadougou, Burkina Faso

**Keywords:** host, pest, plant, refuge, Tephitidae, vegetable

## Abstract

This study was carried out in 3 types of biotopes where vegetable crops are not grown to highlight their contribution to the dynamics of vegetable-infesting flies. To this end, a trapping system based on a sexual attractant, the Cuelure associated with an insecticide was set up in 18 biotopes (6 natural areas, 6 mango orchards, and 6 agroforestry parks) in the regions of Hauts Bassins and Cascades in the South-West of Burkina Faso. During the trapping monitoring, which was done every 2 wk to collect insects captured, fruits present in 3 types of biotopes were sampled and incubated for insect emergence. Ten *Dacus* (Fabricius) [Diptera: Tephritidae] species and *Zeugodacus cucurbitae* (Coquillett) [Diptera: Tephritidae] were trapped in the study area. The predominant species captured was *Z. cucurbitae* (52.93%) followed by *Dacus punctatifrons* (Karsch) [Diptera: Tephritidae] (29.89%) and *Dacus humeralis* (Bezzi) (12.71%). Six tephritid species were emerged from 6 wild fruit species belonging to Cucurbitaceae, Apocynaceae, and Passifloraceae families. Fruit flies were more abundant from Jul to Nov with peaks observed in Aug or Oct depending on the species. *Citrullus colocynthis* L. (Cucurbitaceae)*, Lagenaria* sp. (Cucurbitaceae), *Passiflora foetida* L. (Passifloraceae), and *Passiflora* sp. acted as reservoir host plants of *Dacus ciliatus* (Loew), *Dacus bivittatus* (Bigot), *Dacus vertebratus* (Bezzi) [Diptera: Tephritidae], *D. punctatifrons*, and *Z. cucurbitae*, the major vegetable insect pests in West Africa. The 3 types of biotopes acted as suitable refuge areas of vegetable crop-infesting fruit flies either for the favorable microclimate or for the alternative host plants.

## Introduction

Vegetable crops hold a key position in smallholder agricultural production systems in Africa due to the number of farmers involved, income generation, employment opportunities, and enhancement of food and nutritional security (Dhillon et al. 2005, Rwomushana et al. 2008, Badii et al. 2015b, De Meyer et al. 2015). The sector, however, is constrained by several factors, including insect pests and diseases (Norman 2003, Ekesi and Billah 2006). Tephritid fruit flies (Diptera: Tephritidae) are one of the most economically important group of insects that pose a serious threat to fresh fruit vegetable production in sub-Saharan Africa (Ekesi and Billah 2006, Mwatawala et al. 2006, De Meyer et al. 2013). Fruit flies are responsible for direct and indirect economic damage to many fruit-bearing vegetable crops, particularly cucurbits (White 2006, Vayssières et al. 2007, De Meyer et al. 2012). Direct damage is caused by fruit fly females puncturing the fruit skin for eggs laying (Dhillon et al. 2005, Ekesi and Billah 2006, White 2006). The feeding activities of hatched larvae cause the destruction of host fruits, converting the flesh fruit into an inedible fruit (qualitative damage) (Dhillon et al. 2005, Mwatawala et al. 2006). Infested fruits often fall prematurely resulting in a reduction in fruit production (quantitative damage), increasing insecticide use and production costs. Additionally, indirect losses are associated with quarantine restrictions imposed by importing countries to prevent the entry and establishment of unwanted fruit fly species (Badii et al. 2015a).

The vegetable-damaging fruit fly species in sub-Saharan Africa include both the natives *Dacus* spp. (Fabricius) [Diptera: Tephritidae] and the invasive *Zeugodacus cucurbitae* (Coquillett) [Diptera: Tephritidae] (Vayssières et al. 2007, Ryckewaert et al. 2010, De Meyer et al. 2012, Mokam et al. 2018, Layodé et al. 2020). *Zeugodacus cucurbitae* (formerly named *Bactrocera cucurbitae*) is native to Central Asia (Drew and Hancock 2000), and its first record from the African mainland was restricted to coastal Tanzania and Kenya, and dates back to 1936 (Bianchi and Krauss 1937). In West Africa, it was recorded before 1999 and in the last decade, it remains the most damaging vegetable insect pest and has spread throughout more than 25 countries in sub-Saharan Africa (Mwatawala et al. 2010, Kambura et al. 2018).

In East Africa, *Z. cucurbitae* was associated with 19 and 17 fruit species in Tanzania (Mwatawala et al. 2010) and Kenya (Kambura et al. 2018), respectively. In La Reunion Island, *Z. cucurbitae*, *D. ciliatus* (Loew) [Diptera: Tephritidae], and *Dacus demmerezi* (Bezzi) [Diptera: Tephritidae] are the main insect pests of cucurbitaceous crops and cause up to 90% of yield loss (Vayssières et al. 2008). In Central Africa, Mokam et al. (2018) showed that *Dacus bivittatus* and *Dacus ciliatus* appeared to be the major insect pests associated with cucurbit crop species in Cameroon. Several authors reported the infestation of vegetable crops by fruit flies (Vayssières et al. 2007, 2008, Layodé et al. 2020, Ouédraogo et al. 2020) in West Africa.

Burkina Faso is a Sahelian country located in the heart of West Africa. The climate of the country is characterized by an alternation of 2 distinct seasons: a wet season that extends from May/June to September/October and a dry season, from September/October to May/June. Agriculture occupies more than 80% of the active population. During the dry season, the survival of rural communities depends largely on off-season crops, particularly market gardening. The market gardening sector creates a lot of indirect jobs for traders, transporters, etc. and brings more than 15,254,134 € per year to the national economy with a contribution of more than 3% to Gross Domestic Product (MAH 2011). In addition to its economic value, this sector contributes to food security and nutritional quality by providing vitamins and trace elements to vulnerable groups. However, in recent years, the sector faces many constraints including pressure due to insect pests such as fruit flies.

The main fruit flies encountered in market gardening sites are in sequence *Z. cucurbitae*, *D. ciliatus*, *D. punctatifrons* (Karsch) [Diptera: Tephritidae], *D. bivittatus* (Bigot), and *D. vertebratus* (Bezzi) (Ouédraogo et al. 2020). To cope with fruit flies’ damage, market gardeners use a large range of insecticides to protect vegetable crops (Ouédraogo et al. 2020). These insecticides are purchased in local markets without guarantee of conformity or quality and more than 72% of them are formulated for cotton crops (Naré et al. 2015, Lehmann et al. 2017). This misuse of pesticides affects directly farmers health and can also threaten the health of consumers and environment with risks for pesticide residues in vegetables and, concomitantly, water and soils (Fernandes and Bacci 2010, Naré et al. 2015, Lehmann et al. 2017). It is therefore necessary to develop agroecological management strategies that preserve human and environmental health. To achieve this, it is first necessary to understand the contribution of the vegetation surrounding the market gardening sites to vegetable fruit fly population dynamics. According to Aluja et al. (2012), vegetation surrounding crops and distribution of essential resources (food, shelter, oviposition substrates) strongly influence behavior, distribution, and abundance of the insects.

In fact, in Burkina Faso, vegetable crops are cultivated in environments where mango orchards, agroforestry parks, and natural areas are the common plant formations. Mango orchards and agroforestry parks are cultivated areas where various crops and fruit species are grown. When looking to natural areas, they are mainly home to various wild plant species including trees, shrubs, creepers, and climbers. Wild species could potentially serve as reservoir hosts, which would allow fruit fly populations to persist in space and time, where or when the preferential host fruits are absent (Moquet et al. 2020). De Meyer et al. (2012) reported the infestation of wild fruit species with vegetable fruit flies. The presence of *Z. cucurbitae*, *D. ciliatus*, and *D. vertebratus* on non-cucurbits suggests that they are pests capable of sustaining their populations on alternative host plants even when cucurbits are absent (Kambura et al. 2018).

To the best of our knowledge, no study has yet investigated the dynamic of the fruit flies associated with vegetable in the biotopes where they are not cultivated. The major aim of this study is to determine the role of these biotopes in the dynamic of vegetable crop infesting fruit flies. Specifically, it consists of (i) establishing the seasonal fluctuations of major vegetable crop-infesting fruit flies in these biotopes and (ii) identifying the fruit species that act as reservoir hosts of vegetable crop-infesting fruit flies. Such information would be necessary for the development of sustainable management strategies for tephritid insect pests associated with vegetable crops.

## Materials and Methods

### Study Sites

**Fig. 1.**
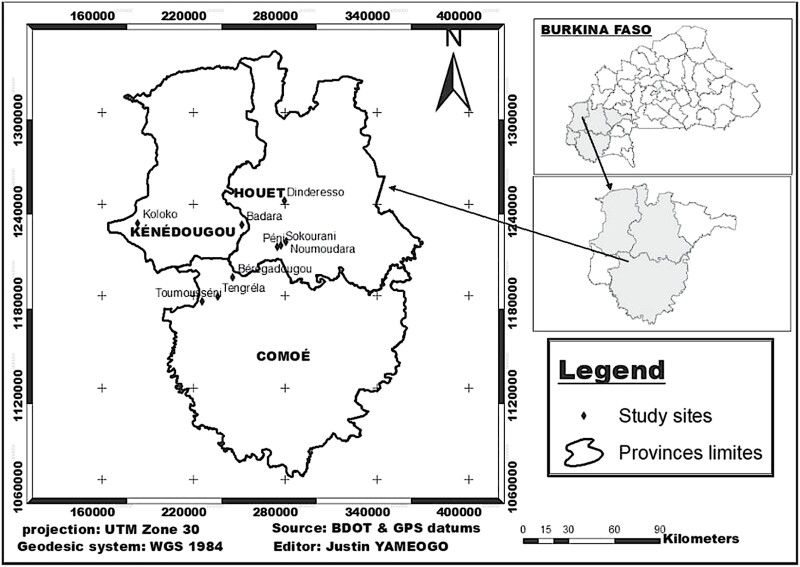
Location of the sites where data were collected from May 2017 to May 2019 in the South-West of Burkina Faso.

Data were collected from 6 municipalities located in the South-West of Burkina Faso (Table 1). In each municipality, fruit fly trapping system and fruit sampling took place in mango orchards, natural areas, and agroforestry parks. In some municipalities such as Bobo-Dioulasso, Koloko, Béréregadougou, and Kourinion, one site was sampled since the 3 plant formations were located in the same village. In the municipality of Péni, 3 sites located in different villages were sampled, with every site hosting a specific plant formation. Mango orchard, natural area, and agroforestry park were located in the villages of Péni, Sokourani, and Noumoudara, respectively. In the municipality of Banfora, mango orchard and natural area were located in Toumousséni, while agroforestry park was located in Tengrela (Fig. 1). Within the same village, a distance of 0.5–3 km separated 2 biotopes.

**Table 1.** GPS coordinates of the 18 biotopes where data were collected in the South-West of Burkina Faso

Provinces	Municipalities	Villages	Type of biotopes	GPS coordinates	
Houet	Péni	Péni	Mango orchard	10°95ʹ21.1″ N	4°46ʹ68.1″ W
		Noumoudara	Agroforestry park	10°96ʹ78.8″ N	4°43ʹ73.1″ W
		Sokourani	Natural area	10°96ʹ36.3″ N	4°44ʹ52.6″ W
	Bobo-Dioulasso	Dindéresso	Natural area	11°21ʹ03.5″ N	4°41ʹ13.3″ W
		Dindéresso	Agroforestry park	11°21ʹ68.1″ N	4°42ʹ30.1″ W
		Dindéresso	Mango orchard	11°21ʹ93.2″ N	4°42ʹ55.7″ W
Comoé	Banfora	Toumousséni	Natural area	10°66ʹ00.4″ N	4ʹ90ʹ42.0″ W
		Toumousséni	Mango orchard	10°65ʹ87.7″ N	4°90ʹ91.8″ W
		Tengrela	Agroforestry park	10°65ʹ56.3″ N	4°87ʹ07.4″ W
	Bérégadougou	Bérégadougou	Natural area	10°81ʹ41.7″ N	4°72ʹ69.2″ W
		Bérégadougou	Mango orchard	10°80ʹ90.3″ N	4°72ʹ69.0″ W
		Bérégadougou	Agroforestry park	10°81ʹ75.0″ N	4°72ʹ63.9″ W
Kénédougou	Kourinion	Badara	Natural area	11°09ʹ32.9″ N	4°62ʹ90.4″ W
		Badara	Mango orchard	11°09ʹ94.9″ N	4°62ʹ39.8″ W
		Badara	Agroforestry park	11°09ʹ30.7″ N	4°62ʹ01.2″ W
	Koloko	Koloko	Natural area	11°09ʹ32.6″ N	5°30ʹ15.9″ W
		Koloko	Mango orchard	11°09ʹ45.1″ N	5°30ʹ40.7″ W
		Koloko	Agroforestry park	11°08ʹ85.7″ N	5°30ʹ75.2″ W

Mango orchards were areas with various varieties of mango (*Mangifera indica* L. [Anacardiaceae]) including improved cultivars (Brooks, Keitt, Kent, Lippens, Springfields, Valencia, Amélie, and Smith) and native ones (Mangot vert and Sabre). In addition, other exotic fruit species namely *Anacardium occidentale* L. (Anacardiaceae), *Psidium guajava* L. (Myrtaceae), *Citrus sinensis* L., and *Citrus limon* L. (Rutaceae) were also encountered in mango orchards.

Agroforestry parks are land-use systems in which woody perennial plants are deliberately conserved in association with cultivation and/or livestock in a dispersed spatial arrangement where there are both ecological and economic interactions between the trees and other components of the system (Baumer 1987, Boffa 2000). In our study area, the shea tree (*Vitellaria paradoxa* C.F. Gaertn. [Sapotaceae]) was the main species representing more than 96% of fruit species encountered in these biotopes and, to a lesser extent, *Parkia biglobosa* (Jacq.) R.Br. ex G.Don [Fabaceae-Mimosoideae], *Lannea microcarpa* (Engl. & K.Krause) [Anacardiaceae], and *Ficus* spp. (Moraceae).

Natural areas included several vegetation types composed of riparian formations (Badara), shrub savannas (Péni, Bérégadougou, and Toumousséni), tree savannas (Koloko), and savanna woodlands (Dindéresso). In the 6 natural areas where fruit were sampled, a total of 99 plant species belonging to 32 families were inventoried. Table 1 presents the GPS coordinates of the 18 biotopes.

**Fig. 2.**
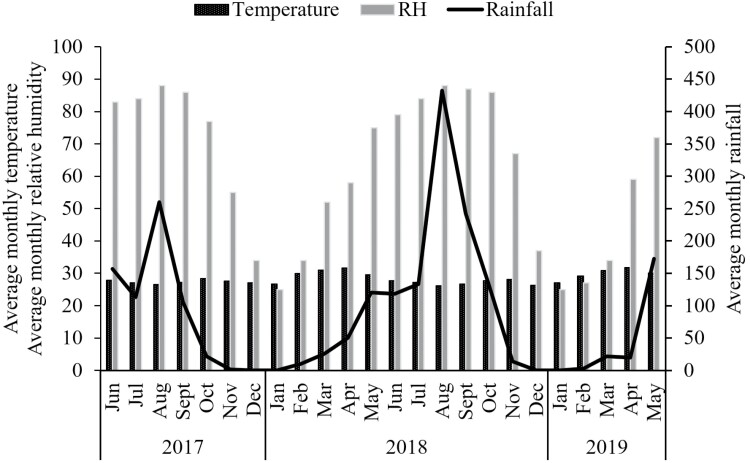
Weather conditions in the study zone during the course of the study: average monthly temperature (°C), average monthly rainfall (mm), and average monthly relative humidity (%).

The weather conditions recorded during the survey in the study zone are presented in Fig. 2. Rains were recorded from May to October with a peak observed in August regardless of the year. Mean monthly relative humidity was lower during the cold period, from December to February (Fig. 2). The average monthly temperature was high from March to May. Weather data were obtained from the National Agency of Meteorology (Burkina Faso).

### Experimental Layout

During this experimentation, sampling was done in mango orchards, agroforestry parks, and natural areas located in the 6 different municipalities. The experimental design was a dispersed block with 3 treatments repeated each, 6 times. The treatments consisted of 3 types of biotopes including natural areas, agroforestry parks, and mango orchards.

### Detection and Monitoring of Fruit Flies in the Tree Types of Biotopes

A total of 72 Tephri traps were hung on tree branches from May 2017 to June 2019 in the 18 biotopes to detect the presence and monitor fruit fly population fluctuations. Since this experimentation targeted vegetable-infesting fruit flies, the sexual attractant, the Cuelure (4-(phydroxyphenyl)-2-butanone acetate), specific to males of *Dacus*, and *Zeugodacus* genera were used as an attractant. In each plant formation, 4 Tephri traps operating with Cuelure associated with an insecticide, the dichlorvos (0,0-dimethyl-0-(2,2-dichloro) phosphate) were used to attract and kill fruit flies. A distance of 50 m was observed between 2 trees where traps were hung. Traps were checked at 2-week intervals and insects caught in each trap were collected and placed in labeled pillboxes. Ethanol diluted at 70% was then added to each pillbox to preserve insects trapped for future identification. Attractants and insecticides containing in each trap were replaced every month during the study.

### Fruit Sampling and Lab Rearing

During trapping periods, fruits found in the 3 types of biotopes were collected for incubation in the laboratory. In natural areas, emphasis was placed on fruits of wild plant species of Apocynaceae, Passifloraceae, and Cucurbitaceae families since they are assumed to be the preferred host fruits of fruit fly species of *Dacus* genus. Depending on fruit availability, 30 fruits were collected on each plant species at each survey date. Fruit samples were packaged in bags and carried to the laboratory for incubation procedures.

Fruit incubation took place in a shady greenhouse implemented at Farako-ba research station (Bobo-Dioulasso, Burkina Faso). Fruit samples were first weighed and placed in plastic containers, the capacity of which varies according to the size of fruits. Large fruits were put in containers that contained a mesh mass that separated the fruits from the sand to make easy the recovery of any pupae formed. The sand contained in each pot was excavated every 5 days, and if necessary, a sieve was used to collect pupae. Pupae were then placed in Petri dishes and stored in a rearing room until adult emergence.

### Identification of Insects

The adult insects captured by the traps and those that emerged from fruits were identified at the entomology laboratory of the research station of Farako-ba (Bobo-Dioulasso, Burkina Faso). With a binocular magnifying glass, the morphological characteristics of the adults were used to classify specimens according to the genera. Identification keys by White (2006), and Virgilio et al. (2014) were used to identify insects at the species level. Some specimens were identified at the Royal Museum for Central Africa, Tervuren, Belgium. The number of specimens of each fruit fly species was recorded per plant formation at each sampling date.

### Data Handling and Statistical Analysis

The number of fruit flies captured in the traps was recorded in each plant formation. The average catch indices expressed as flies per trap per day (FTD) were calculated in the 18 biotopes for the first 3 dominant fruit fly species including *Z. cucurbitae, D. punctatifrons*, and *D. humeralis*. Statistical analyses were performed with R version 3.6.2. Due to the overdispersion of data, the general linear model with quasi-Poisson distribution was used to test the impact of collection dates, study sites, and biotope types and their interactions on the response variable FTD. Linear regression was performed to check the level of influence of the weather conditions (rainfall, temperature, and relative humidity) of the study zone on the FTD of the 3 dominant fruit fly species captured.

Regarding fruit incubations, the infestation index expressed as the number of adult flies that emerged per kg of fruits was calculated for each host fruit species.

## Results

### Detection and Monitoring of Vegetable-Infesting Fruit Flies in the Biotopes

During the 2-year survey, 10 *Dacus* species and *Zeugodacus cucurbitae* (Coquillett) (melon fly) were recorded. The *Dacus* species were *Dacus albiseta* (White and Goodger), *D. bivittatus* (Bigot), *D. ciliatus* (Loew), *D. congoensis* (White), *D. diastatus* (Munro), *D. humeralis* (Bezzi), *D. langi* (Curran), *D. longistylus* (Wiedmann), *D. punctatifrons* (Karsch), and *D. vertebratus* (Bezzi). We noted that 3 species, *D. ciliatus, D. longistylus*, and *D. vertebratus*, were not captured in the traps and 3 other species, *D. albiseta, D. congoensis*, and *D. diastatus*, did not emerge from fruit incubations. *Zeugodacus cucurbitae* was the exotic one and *Zeugodacus* species identified during the survey period.

In total, 34,961 fruit fly specimens were captured, of which *Z. cucurbitae* was the predominant one, with 52.93% of adult caught followed by *D. punctatifrons* (29.89%) and *D. humeralis* (12.71%) (Table 2). With regard to the 3 types of habitats, the highest number of fruit fly adults was caught in mango orchards (41.77%), followed in sequence by agroforestry parks (31.11%) and natural fallows (27.11%). Table 2 presents the distribution and the relative abundance of each fruit fly species captured according to the 3 types of biotopes. The exotic species *Z. cucurbitae* dominated native *Dacus* species in the cultivated areas including mango orchards and agroforestry parks, while the native *Dacus* species were in general more abundant in natural areas (Table 2).

**Table 2.** Distribution and relative abundance of vegetable-infesting fruit flies captured according to the type of biotopes in the South-West of Burkina Faso

Fruit fly species captured	Natural areas		Agroforestry parks		Mango orchards	
	Abundance	Proportions	Abundance	Proportions	Abundance	Proportions
*Z. cucurbitae*	2,846	30.01%	6,125	56.31%	9,533	65.28%
*D. punctatifrons*	4,378	46.18%	2,514	23.11%	3,557	24.36%
*D. humeralis*	1,681	17.73%	1,757	16.15%	1,006	6.89%
*D. albiseta*	136	1.43%	83	0.76%	36	0.24%
*D. bivittatus*	247	2.60%	265	2.43%	345	2.30%
*D. congoensis*	111	1.17%	57	0.52%	29	0.19
*D. diastatus*	77	0.81%	73	0.67%	88	0.6
*D. langi*	5	0.05%	4	0.03%	8	0.05%
Total	9,481	27.11%	10,878	31.11%	14,602	41.77%

### Relative Abundance and Seasonal Fluctuations of Fruit Flies Trapped in the 3 Types of Biotopes in the South-West of Burkina Faso

**Fig. 3.**
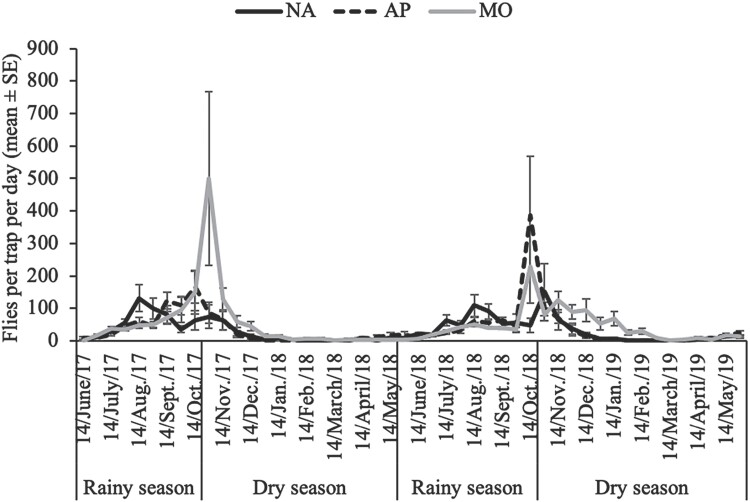
Fluctuations of vegetable-infesting fruit fly populations during the course of study according to the 3 types of biotopes in the South-West of Burkina Faso. NA, natural areas; AP, agroforestry parks; MO, mango orchards.

The dynamics of the average catch indices, expressed as flies per trap per day (FTD) of all fruit fly populations captured during the experimentation, is shown in Fig. 3. The Kruskal–Wallis non-parametric test detected significant differences of FTDs depending on the survey dates (χ^2^ = 453.48, df = 47, *P*-value < 2.2e-16), the study sites (χ^2^ = 37.355, df = 5, *P*-value = 5.084e-07), and the 3 types of habitats (χ^2^ = 20.941, df = 2, *P*-value = 2.836e-05). Fruit flies were more abundant during the rainy season and peaked in August or October depending on the fruit fly species. The majority of fruit fly species were almost absent during the dry and hot months.

### Seasonal Fluctuations of the First 3 Dominant Fruit Fly Species Captured in the 3 Types of Biotopes

**Fig. 4.**
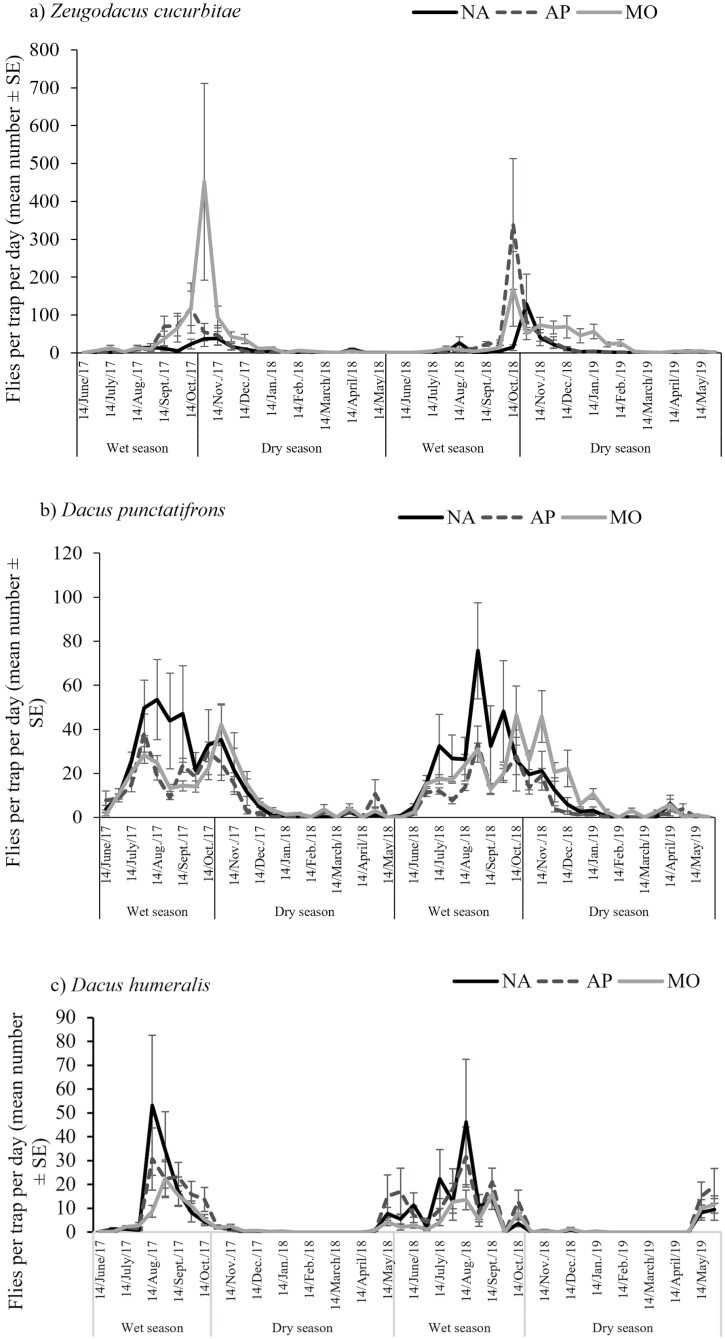
Evolution of the average catch indices (FTD) of the 3 main vegetable-infesting fruit fly species trapped depending on 3 types of biotopes during 2-year surveys in the South-West of Burkina Faso. a) Seasonal fluctuation of *Z. cucurbitae* in the 3 types of biotopes, b) seasonal fluctuation of *D. punctatifrons* in the 3 types of biotopes, and c) seasonal fluctuation of *D. humeralis* in the 3 types of biotopes. NA, natural areas; AP, agroforestry parks; MO, mango orchards.

Figure 4 presents the seasonal fluctuations of the 3 dominant species trapped in the 3 types of biotopes during the entire study.

The FTDs of the exotic and invasive species, *Z. cucurbitae*, varied during the experimentation depending on the survey dates (Fig. 4a). This species was absent in the 3 types of biotopes most of the year especially in natural areas during the dry and hot seasons. Its population density was higher in cultivated areas compared to the natural ones. In fact, its population peaked on 28 October 2017 in mango orchards, while in 2018, the peak was recorded on October 14 in agroforestry parks. Study sites and survey dates exerted a significant effect on *Z. cucurbitae* seasonal fluctuation (Table 3). With respect to the variable interactions, those between study sites and type of biotopes, study sites, and survey dates impacted significantly the seasonal fluctuation of *Z. cucurbitae* (Table 3).

**Table 3.** Effects of different variables and their interactions on the FTDs of the 3 dominant vegetable-infesting fruit fly species trapped during the study in the South-West of Burkina Faso

Variables	D. humeralis			Z. cucurbitae			D. punctatifrons		
	DF	*F*-value	Pr(> *F*)	DF	*F*-value	Pr(> *F*)	DF	*F*-value	Pr(> *F*)
Survey dates	47	3.25	1.27e-11***	47	2.36	2.10e-06***	47	5.49	<2e-16***
Sites	5	36.59	<2e-16***	5	9.55	9.09e-09 ***	5	18.24	<2e-16***
Type of biotopes	2	1.61	0.20	2	2.65	0.07	2	5.30	0.005**
Survey dates × sites	235	3.15	<2e-16***	235	1.60	3.90e-06***	235	1.01	0.43
Survey dates × type of biotopes	94	0.37	1.00	94	0.76	0.94	94	0.57	0.99
Sites × type of biotopes	10	3.23	0.0004***	10	2.95	0.001**	10	15.02	<2e-16***

Signification codes: 0 “***”0.001, “**”0.01, “*”0.05, ‘^.^’ 0.1, ‘’ 1.

The greatest number of flies captured of the most abundant indigenous species, *D*. *punctatifrons*, was from July to November, with the peak observed in August (Fig. 4b). Its FTD was lower in all the 3 types of biotopes during the dry season, from December to May. Natural areas had the highest FTDs of this fruit fly species from July to late October (wet season) while from November to January (cold and dry season) its population was higher in mango orchards. Study sites, survey dates as well as type of biotopes influenced significantly the FTDs of *D. punctatifrons* (Table 3). Only study sites interacted with biotope types to regulate significantly the seasonal abundance of this fruit fly species (Table 3).

*Dacus humeralis* was ranked as the second native fruit fly species trapped during our study. It was present in all the 3 types of biotopes during the rainy season, from May to October and its population was more abundant in natural areas as compared to both cultivated biotopes (Fig. 4c). Its population density reached a peak on 14 August 2017 in natural areas and agroforestry parks, while in mango orchards, it peaked on 28 August. During the 2018 rainy season, the same observations were recorded. No significant effect was exerted by type of biotopes and the interaction between survey dates and biotope types on the capture of *D. humeralis* (Table 3).

### Effects of Climatic Factors on the Population Dynamic of the 3 Dominant Fruit Fly Species in the Study Zone

Results presented in Table 4 show that the average catch indices of *D. punctatifrons* were positively influenced by rainfall, while relative humidity (RH) affected strongly and positively the fluctuations of *D. punctatifrons* in our study conditions. However, maximum temperature exerted a strong negative influence on *D. punctatifrons* FTDs.

**Table 4.** Relationship between weather conditions and population fluctuations of the 3 dominant vegetable crop-infesting fruit fly species trapped from May 2017 to May 2019 in the South-West of Burkina Faso

Variables	*Z. cucurbitae*			D. punctatifrons			D. humeralis		
	Coeff.	*R* ^2^	*P*-value	Coeff.	*R* ^2^	*P*-value	Coeff.	*R* ^2^	*P*-value
Rainfall	−0.006	−0.037	0.689	0.141	0.292	0.003**	0.309	0.595	6.05e-06***
RH	0.004	0.029	0.205	0.038	0.499	6.782e-05***	0.054	0.374	0.0008***
RHMax	0.0028	−0.019	0.463	0.035	0.382	0.0007***	0.055	0.359	0.001**
RHMin	0.0010	−0.039	0.728	0.030	0.445	0.0002***	0.051	0.493	7.784e-05***
Temperature	−0.0002	−0.014	0.419	−0.002	0.285	0.004**	−0.002	0.100	0.072
TempMax	−9.102e-05	−0.043	0.823	−0.0038	0.411	0.0004***	−0.005	0.315	0.002**
TempMin	−9.425e-05	−0.041	0.763	−0.0005	−0.0275	0.541	1.092e-04	−0.045	0.943

Signification codes: 0 “***” 0.001, “**”0.01, “*”0.05, “^.^”0.1, “” 1.

Coeff., linear regression coefficient; *R*^2^, coefficient of determination; rainfall, average monthly rainfall; RH, average monthly relative humidity; RHMax, maximum relative humidity; RHMin, minimum relative humidity; temperature, average monthly temperature; TempMax, maximum temperature; TempMin, minimum temperature.

The population density of *D. humeralis* was strongly and positively affected by both, the average monthly rainfall, and the RH (Table 4). No significant relationship was observed between the average monthly temperature and average catch indices of *D. humeralis* in our study conditions. However, maximum temperature had a negative and significant effect on *D. humeralis* population fluctuations (Table 4).

With regard to the exotic and invasive fruit fly species, *Z. cucurbitae*, no significant influence was observed between its FTDs and the average monthly rainfall, the average monthly temperature, and the average monthly RH (Table 4).

### Alternative Host Plants of Vegetable Damaging Fruit Flies

Among the 29 fruit species infested with fruit flies, only 6 noncultivated plant species belonging to 3 plant families harbored vegetable-infesting fruit flies (Table 5). These latter were all found only in the natural areas. In total, 1,776 fruit fly specimens belonging to 7 *Dacus* species and *Z. cucurbitae* emerged from fruit incubations. The mean number of emerged adults/kg varied according to the fruit species, with wild cucurbit fruit species having the highest number of emerged adults.

**Table 5.** Details of fruit samples infested with fruit flies in the 3 types of biotopes from May 2017 through April 2019 in the South-West of Burkina Faso

Study sites	Biotopes	Plant family	Plant species	Sampling period	Number of fruits	Weight (kg)	Number of pupae	Number of fruit fly adults	Infestation rate (adults/kg)
Badara	Natural area	Annonaceae	*Annona senegalensis* Pers.	April–June	60	0.42	149	–	–
		Amplidaceae	*Cissus populnea* Guill. & Perr.	December–January	250	0.4	6	–	–
		Apocynaceae	*Saba senegalensis* (A. DC.) Pichon	December–January	104	3.59	225	–	–
		Cucurbitaceae	*Lagenaria* sp*.*^a^	August–September	25	4.40	190	148	33.64
		Rubiaceae	*Sarcocephalus latifolius* (Sm.) E.A.Bruce	August–December	300	4.52	617	–	–
		Apocynaceae	*Taccazzea apiculata* ^a^ Oliv.	September–October	125	4.75	179	113	23.79
	Mango orchard	Anacardiaceae	*Mangifera indica* L.	April–July	923	173.22	2,672	–	–
		Myrtaceae	*Psidium guajava* L.	May–June	510	16.04	1,097	–	–
		Rutaceae	*Citrus limon* L.	December–January	30	0.11	1	–	–
	Agroforestry park	Sapotaceae	*Vitellaria paradoxa* C.F. Gaertn.	May–July	1,262	20.10	2,428	–	–
Bérégadougou	Natural area	Annonaceae	*Annona senegalensis* Pers.	May–June	180	2.47	415	–	–
		Apocynaceae	*Landolphia dulcis* (Sabine) Pichon	June–July	126	2.49	109	–	–
		Apocynaceae	*Landolphia heudelotii* A. DC.	May–August	210	3.09	63	–	–
		Cucurbitaceae	*Lagenaria* sp*.*^a^	August–October	30	6.46	343	219	33.90
		Opiliaceae	*Opilia celtidifolia* (Guill. & Perr.) Endl. Ex Walp.	June–September	410	1.99	26	–	–
		Sapotaceae	*Pachystella pobeguiniana* Pierre ex Lecomte	June–July	422	2.12	151	–	–
		Passifloraceae	*Passiflora foetidae* ^a^ L.	October–December	119	1.53	132	116	75.82
		Apocynaceae	*Saba senegalensis* (A. DC.) Pichon	May–January	120	4.65	380	–	–
		Rubiaceae	*Sarcocephalus latifolius* (Sm.) E.A.Bruce	September–October	150	2.47	637	–	–
		Anacardiaceae	*Sclerocarya birrea* (A.Rich.) Hochst.	May–June	300	5.42	151	–	–
		Annonaceae	*Uvaria chamae* P. Beauv.	September–October	50	1.5	59	–	–
	Mango orchard	Anacardiaceae	*Anacardium occidentale* L.	February–May	120	2.25	7	–	–
		Anacardiaceae	*Mangifera indica* L.	April–July	850	194.80	4,504	–	–
	Agroforestry park	Sapotaceae	*Vitellaria paradoxa* C.F. Gaertn.	April–July	2,199	45.22	6,862	–	–
Dindéresso	Natural area	Annonaceae	*Annona senegalensis* Pers.	May–August	270	4.94	286	–	–
		Apocynaceae	*Calotropis procera* (Aiton) R.Br.	January–February	196	2.05	372	356	173.66
		Cucurbitaceae	*Lagenaria* sp.^a^	August–October	24	4	137	109	27.25
		Cucurbitaceae	*Citrullus colocynthis* ^a^ L.	July–September	18	5.35	301	262	48.97
		Apocynaceae	*Landolphia heudelotii* A. DC.	May–July	60	0.55	44	–	–
		Rubiaceae	*Sarcocephalus latifolius* (Sm.) E.A.Bruce	July–November	30	0.6	23	–	–
	Mango orchard	Rutaceae	*Citrus limon* L.	November–December	84	0.75	4	–	–
		Anacardiaceae	*Mangifera indica* L.	May–Jul	542	79.50	2,720	–	–
		Myrtaceae	*Psidium guajava* L.	September–October	120	1.69	54	–	–
	Agroforestry park	Sapotaceae	*Vitellaria paradoxa* C.F. Gaertn.	July–August	765	14.17	533	–	–
Koloko	Natural area	Annonaceae	*Annona senegalensis* Pers.	May–June	210	3.69	1,278	–	–
		Malvaceae	*Cola cordifolia* (Cav.) R.Br.	June–July	85	1.92	101	–	–
		Apocynaceae	*Landolphia heudelotii* A. DC.	May–July	90	1.11	22	–	–
		Passifloraceae	*Passiflora foetidae* ^a^ L.	November–December	91	0.92	103	77	83.69
		Sapindaceae	*Paullinia pinnata* L.	September–November	132	0.52	12	–	–
		Opiliaceae	*Opilia celtidifolia* (Guill. & Perr.) Endl. Ex Walp.	June–August	270	1.58	5	–	–
		Cucurbitaceae	*Citrullus colocynthis* ^a^ L.	July–September	6	1.85	157	89	48.11
		Moraceae	*Ficus sycomorus* L.	September–December	150	1.83	18	–	–
		Apocynaceae	*Saba senegalensis* (A. DC.) Pichon	May–December	30	0.52	148	–	–
		Rubiaceae	*Sarcocephalus latifolius* (Sm.) E.A.Bruce	July–October	450	8.32	247	–	–
		Anacardiaceae	*Spondias mombin* L.	October–November	422	4.46	183	–	–
		Myrtaceae	*Syzygium guineense* (Willd.) DC.	April–May	182	1.85	19	–	–
		Annonaceae	*Uvaria chamae* P. Beauv.	September–October	152	3.85	50	–	–
	Mango orchard	Anacardiaceae	*Anacardium occidentale* L.	Mar–May	60	1.30	3	–	–
		Rutaceae	*Citrus limon* L.	November–January	90	0.95	6	–	–
		Rutaceae	*Citrus sinensis* L.	November–January	40	0.85	3	–	–
		Anacardiaceae	*Mangifera indica* L.	April–July	714	161.62	3,947	–	–
	Agroforestry park		*Vitellaria paradoxa* C.F. Gaertn.	May–July	1,226	22.72	2,270	–	–
Sokourani	Natural area	Annonaceae	*Annona senegalensis* Pers.	May–June	66	0.57	311	–	–
		Apocynaceae	*Calotropis procera* ^a^ (Aiton) R.Br.	January–February	122	0.77	165	102	132.47
		Amplidaceae	*Cissus populnea* Guill. & Perr.	September–November	126	0.23	11	–	–
		Passifloraceae	*Passiflora* sp.^a^	October–December	57	0.75	72	59	78.67
		Rubiaceae	*Sarcocephalus latifolius* (Sm.) E.A.Bruce	July–November	603	11.95	2,525	–	–
		Loganiaceae	*Strychnos innocua* Delile	June–August	123	11.40	284	–	–
		Loganiaceae	*Strychnos spinosa* Lam.	June–August	103	9.65	90	–	–
Péni	Mango orchard	Anacardiaceae	*Mangifera indica* L.	April–June	443	43.76	865	–	–
		Myrtaceae	*Psidium guajava* L.	September–October	110	1.55	1	–	–
Noumoudara	Agroforestry park	Sapotaceae	*Vitellaria paradoxa* C.F. Gaertn.	May–July	2,980	62.57	1,1832	–	–
Toumousséni	Natural area	Annonaceae	*Annona senegalensis* Pers.	May–August	270	5.78	789	–	–
		Malvaceae	*Cola cordifolia* (Cav.) R.Br.	June–July	60	1.2	81	–	–
		Passifloraceae	*Passiflora* sp*.*^a^	October–December	90	1.45	87	79	54.48
		Apocynaceae	*Landolphia heudelotii* A. DC.	May–July	60	0.85	1	–	–
		Apocynaceae	*Saba senegalensis* (A. DC.) Pichon	May–December	90	1.57	209	–	–
		Cucurbitaceae	*Citrullus colocynthis* ^a^ L.	July–September	13	3.05	192	154	50.49
		Rubiaceae	*Sarcocephalus latifolius* (Sm.) E.A.Bruce	July–November	420	7.80	2,063	–	–
	Mango orchard	Anacardiaceae	*Anacardium occidentale* L.	March–May	90	1.70	6	–	–
		Rutaceae	*Citrus sinensis* L.	November–December	71	2.30	8	–	–
		Anacardiaceae	*Mangifera indica* L.	May–July	1,104	272.55	9,026	–	–
Tengrela	Agroforestry park	Sapotaceae	*Vitellaria paradoxa* C.F. Gaertn.	July–August	1,061	18.39	965	–	–

Type of biotopes: habitats where data were collected in each site, plant species: fruit species name followed by author, fruits: total number of fruits collected per plant species, sampling period: period of availability of fruits at physiological maturity, weight: total weight of fruit sampled per plant species in each type of biotopes, pupae: total number of pupae obtained, number of fruit fly adults: total number of adults of vegetable fruit flies emerged, infestation rate: number of fruit fly adults/kg fruit. “–” indicates no vegetable damaging fruit fly adult emerged.

^a^Plant species infested with vegetable damaging fruit flies.

**Fig. 5.**
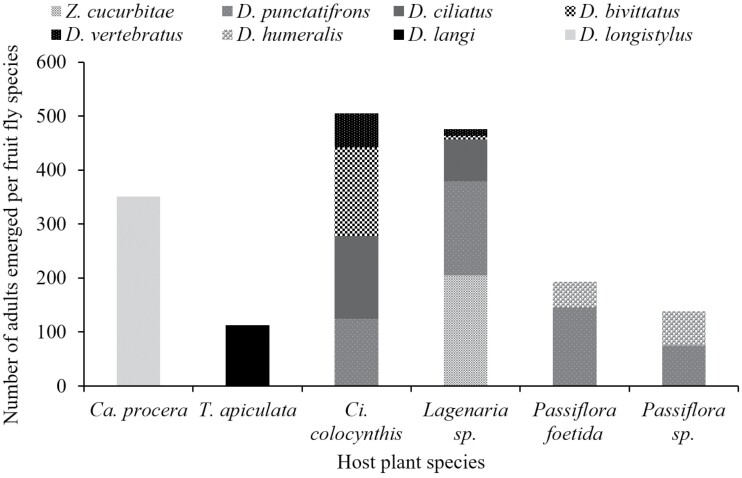
Fruit fly species associated with noncrop plant species in the South-West of Burkina Faso.

Figure 5 shows the diversity of fruit flies that emerged per host plant and the abundance of each fruit fly species. The Afrotropical fruit fly species, *D. punctatifrons* was the most numerous species that emerged and was associated with the highest number of fruit species (4 fruit species). It shared fruits of the Passifloraceae family with *D. humeralis*. The exotic and invasive species, *Z. cucurbitae* was associated with a single fruit species, *Lagenaria* sp. It shared this host with 4 native *Dacus* species. Two indigenous species including *Dacus langi* and *D. longistylus* emerged from a single fruit species, respectively *T. apiculata* and *C. procera*. Cucurbit species harbored the highest number of fruit fly species, up to 4 species emerged from *C. colocynthis* and 5 from *Lagenaria* sp.

## Discussion

Among the 11 fruit fly species recorded during the survey period, 10 were of the genus *Dacus* and 1 of the genus *Zeugodacus. Dacus ciliatus, D. longistylus*, and *D. vertebratus* were not captured with the sexual attractant Cuelure, while *D. albiseta*, *D. congoensis*, and *D. diastatus* did not emerge from any host fruit detected in the current study. The economic important species recorded were *Z. cucurbitacae*, *D. bivittatus* and *D. punctatifrons*, *D. ciliatus*, and *D. vertebratus*. These results suggest that trapping and fruit incubations are complementary methods in the establishment of fruit fly diversity in a given area. These findings are in accordance with reports by Zida et al. (2020a, 2020b) who showed the presence of all these tephritid species in the study zone. Among the tephritid species, *Z. cucurbitae*, *D. ciliatus*, *D. vertebratus*, *D. bivittatus* (Dhillon et al. 2005, Mwatawala et al. 2006, De Meyer et al. 2015), and *D. punctatifrons* (Mokam et al. 2018) were found to be the major vegetable damaging fruit fly species in sub-Saharan Africa.

Six non-crop plant species belonging to the Cucurbitaceae, Apocynaceae, and Passifloraceae families were infested by fruit flies. Studies by White (2006) and Mwatawala et al. (2006) showed that the *Dacus* species infested creepers and climbers of either Cucurbitaceae, Apocynaceae, or Passifloraceae. Among the host plant species recorded, 4 species including *C. colocynthis*, *Lagenaria* sp., *P. foetida*, and *Passiflora* sp. can be considered as alternative hosts of vegetable-damaging fruit flies. When looking at the host fruits of the Apocynaceae family recorded in our study, they seem to be infested with fruit fly species of minor interest. This is the first time that the infestation of the fruit of *T. apiculata* with *D. langi* is recorded. This fruit fly species was identified in some African countries (N’dépo et al. 2009, Vayssières et al. 2010, 2015, Virgilio et al. 2011) although, its host range was not mentioned. *Dacus longistylus* seems to attack only fruits of *C. procera*. We did not find studies showing its association with vegetable crops. Thus, *D. langi* and *D. longistylus* are believed to be non-pest fruit fly species (Doorenweerd et al. 2018).

All alternative host plants identified in the current study were only found in natural areas. Such a result could be explained by the fact that host fruits identified are wild plant species, while mango orchards and agroforestry parks shelter mainly exotic cultivated fruit species like mango, guava, and *Citrus* spp. and indigenous semi-domesticated plant species like shea tree, respectively.

From all tephritid species that emerged, *D. punctatifrons* had the largest host range with 4 infested plant species. The infestation of *P. foetida* by *D. punctatifrons* is reported by White (2006). *Dacus humeralis* was the only attacked plant species of the Passifloraceae family, confirming a report by Mwatawala et al. (2006). The major vegetable insect pest species, natives to Africa such as *D. ciliatus*, *D. bivittatus*, and *D. vertebratus*, attacked at least 2 fruit species, while the exotic *Z. cucurbitae* attacked only *Lagenaria* sp. These observations concur with those by Zida et al. (2020b) who indicated that the native species *Ceratitis* spp. dominated the exotic species *B. dorsalis* on indigenous fruit species. During the current study, 3 *Dacus* species, *D. albiseta, D. congoensis*, and *D. diastatus*, were not associated with any host plant species. The presence of any fruit fly species in an ecosystem does not necessarily mean that it is associated with a fruit species (Aluja and Mangan 2008).

Neither *Dacus* nor *Zeugodacus* species emerged from cultivated fruit species including mango, guava, cashew, and *Citrus* spp. during our 2-year survey. Similar results were reported by Badii et al. (2015b) and Gomina et al. (2020) who did not find any *Dacus* species infesting mango, guava, and cashew, respectively, in Northern Ghana and in Togo Republic. However, our findings did not confirm those of Vayssières et al. (2007); Vayssières et al. (2010); N’dépo et al. (2013); Vayssières et al. (2015) who reported the infestation of mango by *Z. cucurbitae*, *D. bivittatus*, *D. punctatifrons*, and *D. vertebratus*, cashew, *Citrus* spp., and *Annona senegalensis* by *Z. cucurbitae* in West Africa.

In our study conditions, the 3 major fruit fly species captured during this study experienced different fluctuation patterns.

The invasive and exotic species, *Z. cucurbitae* was more abundant from August to December and peaked in October at the beginning of the dry season. Moreover, weather conditions including rainfall, temperature, and RH did not affect significantly *Z. cucurbitae* seasonal fluctuations. Similar observations were reported by Gnanvossou et al. (2017) in Benin. Therefore, we can conclude that the abundance of this exotic species is mainly regulated by biotic factors, especially host fruit availability. In the South-West of Burkina Faso, vegetable crops of Cucurbitaceous family are grown at the end of the rainy season from September to December explaining the peak of *Z. cucurbitae* during this period in the biotopes surrounding market gardening sites. *Zeugodacus cucurbitae* was the only exotic species recorded and was more abundant than the native *Dacus* species, especially in the cultivated areas. The dominance of *Z. cucurbitae* over the native *Dacus* species was reported in Tanzania and Benin (Mwatawala et al. 2006, Gnanvossou et al. 2017). These observations concur with findings of several studies carried out in sub-Saharan Africa, which showed that the exotic species, *Bactrocera dorsalis* Hendel, dominated the indigenous species of the genus *Ceratitis* in plant formations (Mwatawala et al. 2009, Vayssières et al. 2015, Gnanvossou et al. 2017, Bota et al. 2018, Zida et al. 2020a).

The density of the population of the 2 native species, *D. punctatifrons* and *D. humeralis*, was found to be higher from July to mid-September and peaked in August depending on the biotopes. In fact, rainfall and RH exerted positive influence on the FTDs of *D. punctatifrons* and *D. humeralis* explaining their abundance observed in August, the month that recorded the highest rainfall and RH during the study. Therefore, the weather conditions prevailing in August coupled with the fruiting of wild cucurbitaceous fruit species can be associated with higher native *Dacus* species FTDs recorded during the rainy season. Geurts et al. (2012) reported that host availability and climatic differences seem to be the determining factors to explain the differences in fruit fly occurrence and abundance in time and space. Indeed, in the study zone, natural areas are home to wild host species which were mainly infested by native *Dacus* fruit fly species. It is well known that host fruits play a major role in the distribution and abundance of fruit fly species (Mwatawala et al. 2006, Vayssières et al. 2015, Zida et al. 2020b, Facon et al. 2021). In contrast, temperature had a negative influence on the seasonal abundance of the 2 fruit fly species. This could explain the lower *D. punctatifrons* FTDs and the absence of *D. humeralis* (December to April) during the hot and dry season when temperature was higher.

The FTDs of native *Dacus* species were higher in natural areas as compared to cultivated areas. In addition, *Dacus punctatifrons* dominated *Z. cucurbitae* only in natural areas. Previous studies reported that tephritid species of sub-Saharan African origin are well adapted to the climatic conditions and indigenous fruit species than the exotic fruit fly species (Carroll et al. 2002, Mwatawala et al. 2009, Geurts et al. 2012, 2014, Zida et al. 2020b).

Survey period and study sites impacted significantly fruit fly population fluctuations. That was because the majority of fruit fly species observed appeared in the 3 types of biotopes with the initiation of the rainy season. In fact, the majority of fruit fly species were absent in the 3 types of biotopes from February to April when little or no rainfall was recorded. Also, the composition and the structure of the biotopes vary depending on the localities.

Significant variation in fruit fly population density was observed between the 3 types of biotopes. In fact, the FTDs were higher in mango orchards and agroforestry parks as compared to the natural areas where wild host fruits are found. Such a result could be explained by the fact that mango orchards and agroforestry parks are cultivated areas, whereas the persistence of tree foliage in these biotopes constituted a suitable microclimate for fruit fly adults during the dry and hot months. Dhillon et al. (2005) demonstrated that during the hot and dry season, the fruit fly adults take shelter under humid and shady places and feed on honeydew of aphids infesting the fruit trees. According to Bateman (1972) and Matanmi (1975), in the Dacini tribe, mating and food intake take place essentially on non-host plants, which are not involved either in the egg laying or in the development of larvae. According to Aluja et al. (2012), vegetation surrounding crops, and distribution of essential resources (food, shelter, and oviposition substrates) strongly influence behavior, distribution, and abundance of the insects.

The current study on the population dynamics of vegetable crop-infesting fruit flies in the biotopes where they are not cultivated is the first of its kind undertaken in Burkina Faso in terms of time and type of biotopes considered. In our study zone, vegetable crops especially Cucurbit crops are grown at the end of the rainy season from September to December. Wild cucurbit species that are available from July to September act as alternative host fruits of vegetable-infesting fruit flies during this period. Moquet et al. (2020) reported that wild species could potentially serve as reservoir hosts, which would allow fruit fly populations to persist in space and time, where or when the preferred host plant species are not available. This 2-year survey reveals the importance of the common plant formations surrounding market gardening sites in the dynamic of vegetable-infesting fruit flies in Burkina Faso.
